# A case of pulmonary primary MALT lymphoma with distinctive bronchoscopic findings

**DOI:** 10.1002/rcr2.1364

**Published:** 2024-05-01

**Authors:** Hiroyuki Miura, Jun Miura, Shinichi Goto, Tomoko Yamamoto

**Affiliations:** ^1^ Department of Thoracic Surgery Akiru Municipal Medical Centre Tokyo Japan; ^2^ Department of Surgery Kyorin University School of Medicine Tokyo Japan; ^3^ Department of Respirology Akiru Municipal Medical Centre Tokyo Japan; ^4^ Department of Pathology Tokyo Women's Medical University Tokyo Japan

**Keywords:** IRTA‐1, MALT lymphoma, mucosa‐associated lymphoid tissue (MALT), pulmonary extranodal marginal zone lymphoma of mucosa‐associated lymphoid tissue, pulmonary MALT

## Abstract

Mucosa‐associated lymphoid tissue (MALT) is a low‐grade lymphoma, but cases in which it has transformed into a high‐grade lymphoma have been reported, necessitating an accurate diagnosis. The patient was a 79‐year‐old nonsmoking Japanese female with history of ocular sarcoidosis. A computed tomography scan of her chest revealed a 35‐mm nodule in the left S1 + 2, contiguous with the lymph nodes. Additional nodules were observed around the left B5 and B10a. Bronchoscopy revealed stenosis caused by a white, glossy, elevated lesion with angiogenesis at the orifice of the left upper lobe bronchus. The biopsy specimen demonstrated the dominance of lymphoid cells and tested positive for CD20, CD79a, Bcl‐2, and IRTA‐1, which is consistent with the findings in MALT lymphoma. Therefore, in the presence of multiple infiltrative shadows along the bronchi with glossy elevated lesions without necrosis on bronchoscopy, it is important to consider MALT lymphoma as a differential diagnosis.

## Graphical abstract

This article is a case of pulmonary primary MALT lymphoma with characteristic bronchoscopy findings. Immunochemical staining of IRTA‐1 was especially useful for diagnosis.

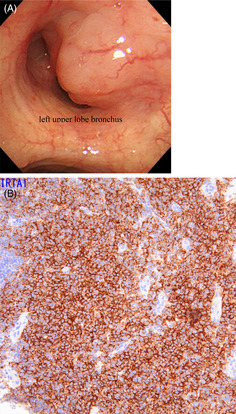

## INTRODUCTION

Pulmonary mucosa‐associated lymphoid tissue (MALT) lymphoma is rare, accounting for less than 0.5% of all primary malignant lung tumours. However, it accounts for 70%–90% of all primary lung malignant lymphomas.^1^ The rate of proper diagnosis for this tumour, other than surgery, is low. The sensitivity of bronchial and transbronchial biopsies in detecting MALT lymphoma have been reported to be 31% and 88%, respectively.^2^ Herein, we report a case of pulmonary MALT lymphoma with characteristic bronchoscopic findings that was accurately diagnosed using immunostaining.

## CASE REPORT

**FIGURE 1:**
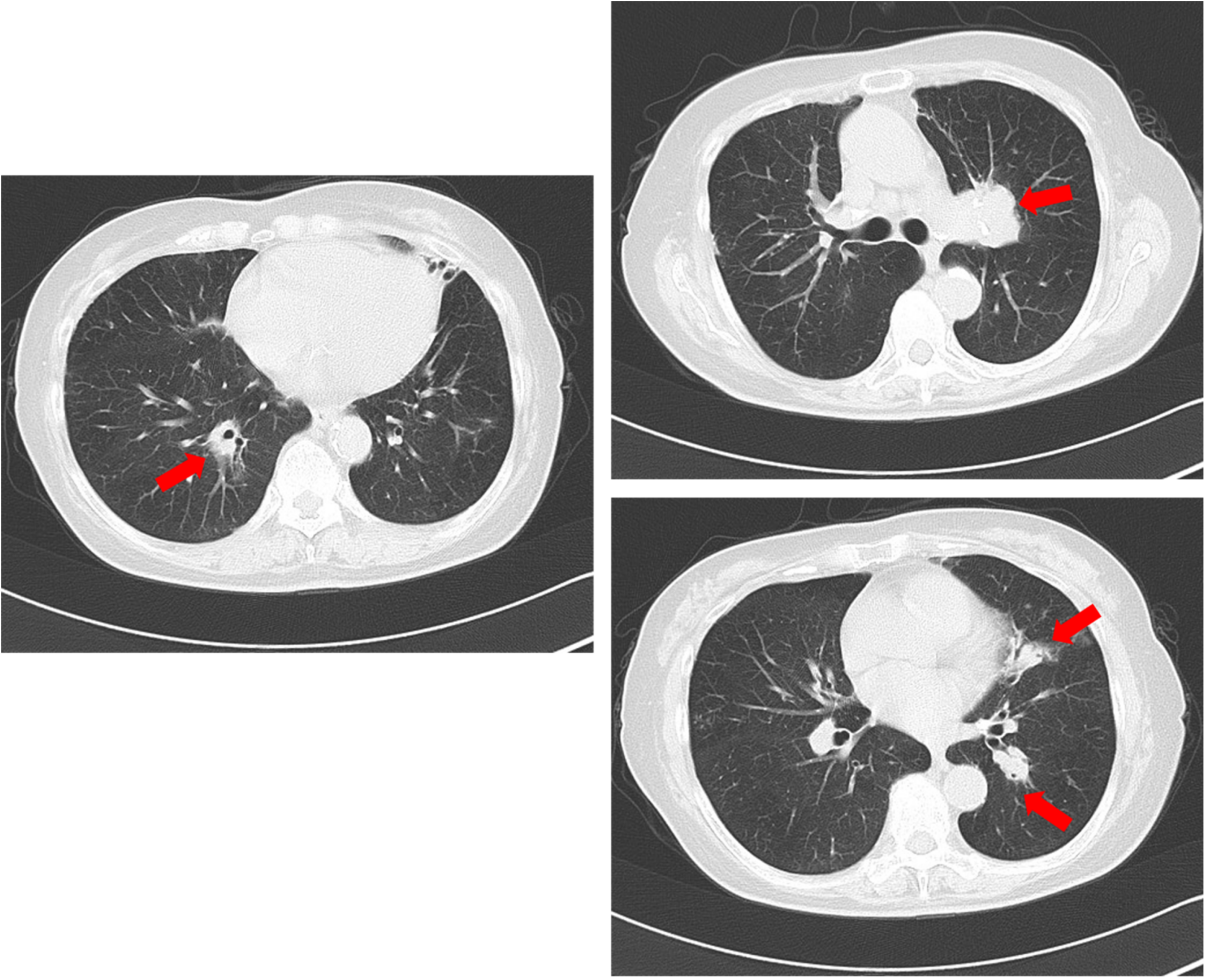
Chest computed tomography scan showing a 35‐mm nodule in her left S1 + 2 contiguous with the lymph nodes (A) and nodules around the right B10 (B), left B5 and B10a (C).

**FIGURE 2:**
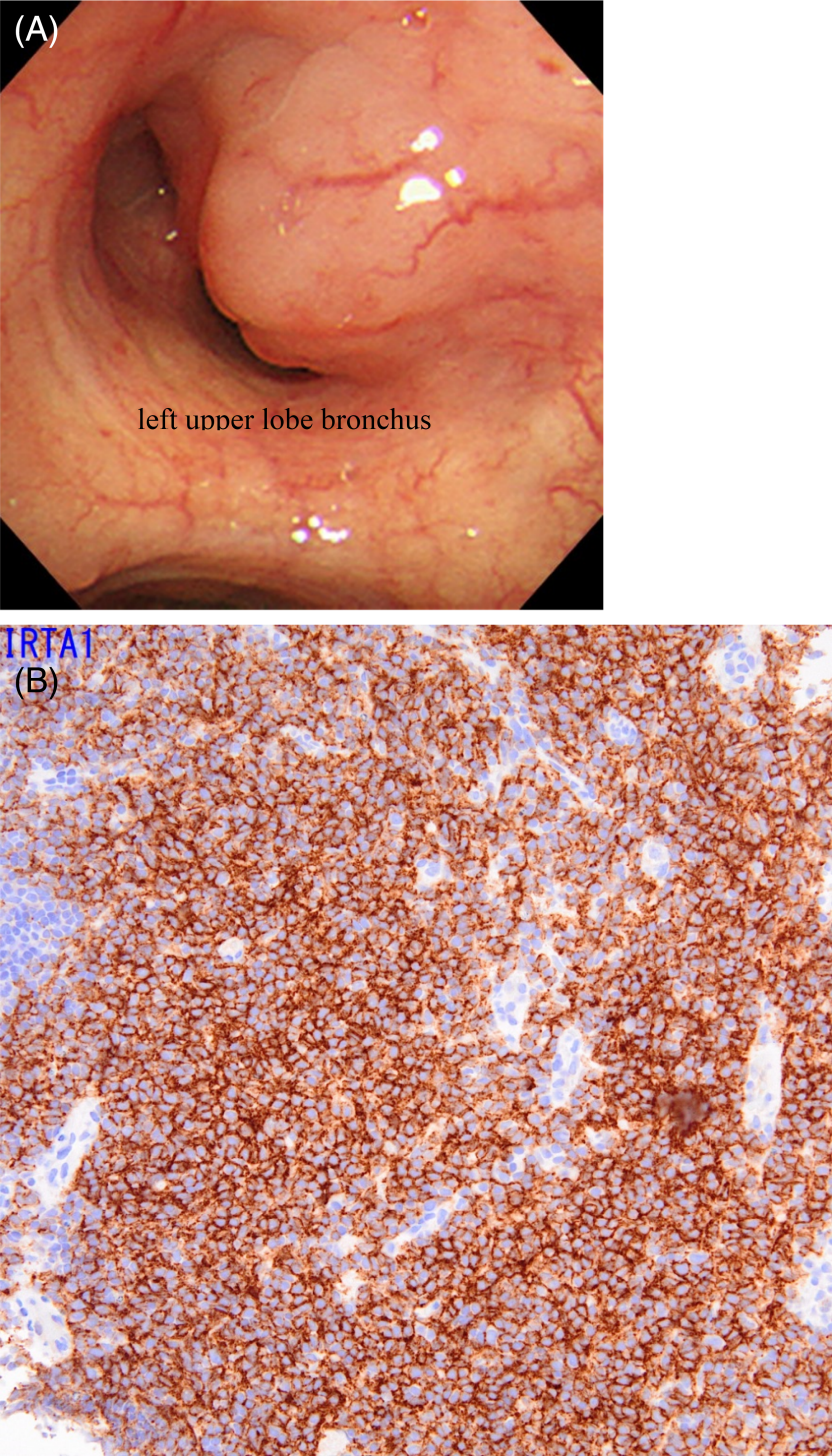
(A) Bronchoscopic images demonstrating mixed stenosis (mucosal and submucosal type) caused by a white, glossy, elevated lesion with angiogenesis at the orifice of the left upper lobe bronchus, which had a smooth surface (A). After taking biopsies, the upper division bronchus was obstructed by the mass (B). (B) (Immunohistochemical staining ×20) The biopsy tissue is positive for IRTA‐1.

A 79‐year‐old Japanese female patient presented with an abnormal shadow on chest x‐ray during an annual checkup. She had always been a nonsmoker, and her family history was unremarkable. The patient had previously been treated for hypertension and hyperlipidemia. She also had a history of ocular sarcoidosis. The chest x‐ray showed the presence of an ill‐defined nodule, approximately 35 mm in diameter, in the left upper lung field. Computed tomography (CT) scan of her chest revealed a 35‐mm nodule in the left S1 + 2 contiguous with the lymph nodes. Additional nodules were detected around the right B10, left B5 and left B10a (Figure 1). Positron emission tomography scan revealed accumulation of fluorodeoxyglucose in the left lung tumour, and in the hilum, mediastinum, and right subclavian lymph nodes. Blood samples showed high levels of soluble interleukin 2 receptor (sIL‐2R) and anti‐Epstein–Barr nuclear antigen IgG. The hemogram and renal and hepatic functions were normal. The levels of tumour markers including carcinoembryonic antigen (CEA), cytokeratin 19 fragment (CYFRA21‐1), and pro‐gastrin releasing peptide (proGRP) were within normal limits. Bronchoscopy revealed mixed stenosis (epitherial and subepitherial type) caused by a white glossy elevated lesion with angiogenesis at the orifice of the left upper lobe bronchus, which had a smooth surface. The upper division of the bronchus was obstructed by a similar tumour existing the orifice of the left upper lobe bronchus (Figure 2A). The specimen biopsied with forceps revealed a dominance of lymphoid cells and tested positive for CD20, CD79a, Bcl‐2, and immunoglobulin superfamily receptor translocation‐associated 1 (IRTA‐1, Figure 2B). Based on these findings, a diagnosis of MALT lymphoma was made. The Ki‐67 labeling index was 20%–30%. No area of transformation to the high‐grade lymphoma was observed. She was referred to a haematologist at another hospital.

## DISCUSSION

The imaging findings of pulmonary MALT lymphoma are diverse, and include nodules, ground‐glass opacities, and bronchiectasis. In our case, the nodule in the lung field was suspected to be a MALT lymphoma for the following reasons: It engulfed the lymph nodes around the bronchi, enlarged and invaded the bronchi, and the presence of multiple lesions. It has been reported that 79% of pulmonary MALT lymphomas consist of multiple lesions and that 66% of the cases have bilateral lesions.^3^ Chest CT of our patient also revealed multiple nodal shadows along the bronchi on both sides.

The pathological findings of MALT lymphoma are diverse and can include reactive lymphoid follicles, diffuse infiltration of centrocyte‐like lymphocytes, lymphoepithelial lesions, and differentiation into plasma cells.^1^ One reason for the poor diagnostic value of biopsy specimens is that the immunostaining results vary depending on the bronchial epithelium and infiltrated inflammatory cells. IRTA‐1 expression is strongly correlated with MALT lymphoma and is extremely useful as an adjunct for the diagnosis of MALT lymphoma.^4^ Chronic inflammation has been reported to cause MALT lymphoma.^5^ The bronchus‐associated lymphoid tissue (BALT) which may be origin of the MALT lymphoma is nonprevalent in healthy adults. The continuous inhaled antigenic stimulations as well as the local production of interleukin‐4 and cytokines promote BALT development, which may give rise to MALT lymphoma. In the case of the present patient, her history of sarcoidosis and Epstein–Barr virus infection were considered possible contributors to the development of MALT lymphoma.

MALT lymphoma is a low‐grade lymphoma, but its transformation into a high‐grade lymphoma has been reported,^6^ and accurate diagnosis is necessary. The presence of multiple infiltrative shadows along the bronchi on images combined with bronchoscopy findings of glossy, elevated lesions without necrosis, should raise the possibility of MALT lymphoma as a differential diagnosis.

## AUTHOR CONTRIBUTIONS

Dr Hiroyuki Miura and Dr Shinichi Goto helped in the conception and design of the work and the acquisition and analysis or interpretation of data for the work. Dr Jun Miura drafted the work and revised it critically for important intellectual content. Dr. Yamamoto diagnosed this tumour pathologically. All authors contributed to the final version of this manuscript and approved it to be published.

## CONFLICT OF INTEREST STATEMENT

None declared.

## ETHICS STATEMENT

Appropriate written informed consent was obtained for publication of this case report and accompanying images.

## Data Availability

The data that support the findings of this study are available on request from the corresponding author. The data are not publicly available due to privacy or ethical restrictions.
